# Malaysian brown macroalga *Padina australis* mitigates lipopolysaccharide-stimulated neuroinflammation in BV2 microglial cells

**DOI:** 10.22038/IJBMS.2023.67835.14842

**Published:** 2023

**Authors:** Kogilavani Subermaniam, Sze Yuen Lew, Yoon Yen Yow, Siew Huah Lim, Wing Shan Yu, Lee Wei Lim, Kah Hui Wong

**Affiliations:** 1 Department of Anatomy, Faculty of Medicine, Universiti Malaya, 50603 Kuala Lumpur, Malaysia; 2 Sungai Buloh Training Institute of Ministry of Health Malaysia, Jalan Hospital, 47000 Sungai Buloh, Selangor, Malaysia; 3 Department of Biological Sciences, School of Medical and Life Sciences, Sunway University, 47500 Bandar Sunway, Selangor Darul Ehsan, Malaysia; 4 Department of Chemistry, Faculty of Science, Universiti Malaya, 50603 Kuala Lumpur, Malaysia; 5 Neuromodulation Laboratory, School of Biomedical Sciences, Li Ka Shing Faculty of Medicine, The University of Hong Kong, 21 Sassoon Road, Pokfulam, Hong Kong Special Administrative Region, China

**Keywords:** Brown algae, BV2 microglial, Cytokines, Major compounds, Neuroinflammation, Oxidative damage

## Abstract

**Objective(s):**

Neuroinflammation and microglial activation are pathological features in central nervous system disorders. Excess levels of reactive oxygen species (ROS) and pro-inflammatory cytokines have been implicated in exacerbation of neuronal damage during chronic activation of microglial cells. *Padina australis*, a brown macroalga, has been demonstrated to have various pharmacological properties such as anti-neuroinflammatory activity. However, the underlying mechanism mediating the anti-neuroinflammatory potential of *P. australis* remains poorly understood. We explored the use of Malaysian *P. australis* in attenuating lipopolysaccharide (LPS)-stimulated neuroinflammation in BV2 microglial cells.

**Materials and Methods:**

Fresh specimens of *P. australis* were freeze-dried and subjected to ethanol extraction. The ethanol extract (PAEE) was evaluated for its protective effects against 1 µg/ml LPS-stimulated neuroinflammation in BV2 microglial cells.

**Results:**

LPS reduced the viability of BV2 microglia cells and increased the levels of nitric oxide (NO), prostaglandin E_2_ (PGE_2_), intracellular reactive oxygen species (ROS), inducible nitric oxide synthase (iNOS), cyclooxygenase-2 (COX-2), tumor necrosis factor-alpha (TNF-α), and interleukin-6 (IL-6). However, the neuroinflammatory response was reversed by 0.5–2.0 mg/ml PAEE in a dose-dependent manner. Analysis of liquid chromatography-mass spectrometry (LC-MS) of PAEE subfractions revealed five compounds; methyl α-eleostearate, ethyl α-eleostearate, niacinamide, stearamide, and linoleic acid.

**Conclusion:**

The protective effects of PAEE against LPS-stimulated neuroinflammation in BV2 microglial cells were found to be mediated by the suppression of excess levels of intracellular ROS and pro-inflammatory mediators and cytokines, denoting the protective role of *P. australis* in combating continuous neuroinflammation. Our findings support the use of *P. australis* as a possible therapeutic for neuroinflammatory and neurodegenerative diseases.

## Introduction

Emerging evidence suggests that neuroinflammation plays an important role in the onset and progression of neurodegenerative diseases. The neuroinflammatory response is mediated by cytokines, chemokines, reactive oxygen species (ROS), and secondary messengers produced by microglia, astrocytes, endothelial cells, peripherally derived T cells, macrophages, and dendritic cells, leading to physiological, biochemical, and psychological consequences (1). Microglia are the resident macrophages of the central nervous system (CNS) and make up approximately 5% to 12% of the total cell population in the CNS. Microglia have been observed to colonize the brain as early as embryonic day 9.5 (E9.5) of development before the emergence of neurons and other glia, and subsequently proliferate and persist throughout one’s lifetime (2). During development, microglia play an active phagocytic role in mediating synaptic pruning and neuronal circuit formation (3). Chronic microglial activation can be triggered by either a single pathogenic stimulus or exposure to multiple stimuli, resulting in cumulative neuronal loss over time. In CNS diseases, reactive microgliosis and ROS have been found to exacerbate the chronic deleterious activation of microglial (4).

Lipopolysaccharide (LPS) is an endotoxin derived from the outer membrane of Gram-negative bacteria. It is primarily recognized by the CD14/Toll-like receptor 4 (TLR-4) receptor complex, which is expressed on microglia and astrocytes. TLR-4 signaling mediates autoimmune responses and neuroinflammation in neurodegenerative diseases (5). Activated microglia generate massive amounts of ROS, acquire a pro-inflammatory cellular profile, and release pro-inflammatory cytokines. Indeed, chronic neuroinflammation involves long-standing activation of microglia and subsequent sustained release of inflammatory mediators resulting in increased oxidative and nitrosative damage (6). 

**Figure 1:**
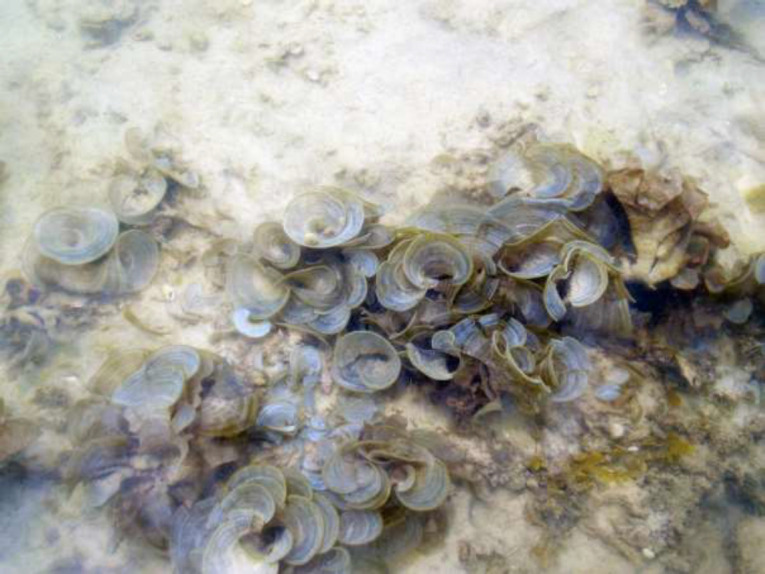
An underwater view of *Padina australis* Hauck, 1887 at Cape Rachado, Port Dickson, Negeri Sembilan, Malaysia

Marine algae are rich in secondary metabolites with therapeutic potential, including phlorotannins, alginates, fucoidan, sargaquinoic acid, sulfated polysaccharides, and carotenoids, which are not commonly found in terrestrial plants (7-11). One such seaweed is brown macroalga, *Padina australis* Hauck 1887 (Figure 1), which can be found in the *slow-moving *and shallow *waters *across the tropical and subtropical regions. Brown macroalgae play an essential role in coastal ecosystems including acting as structures, trapping nutrients and carbon, producing oxygen, and generating biomass (12-15). *P. australis* can be found in lower intertidal to deep subtidal zones along the moderately wave-exposed shorelines of coastal regions. It can be spotted *anchored *to a solid *substrate* by a discoid holdfast on coral rubble, reef flats, tidepools, and fishing nets (16). *P. australis* possesses pharmacological properties such as anti-oxidant (17-19), anti-neuroinflammatory (17), antimicrobial (20, 21), antiacetylcholinesterase (17, 18), anticancer (22), antiviral (23), antiangiogenic (24), and neurotrophic (25) activity. Our recent findings showed that *P. australis* promoted antidepressant-like effects in an *in vitro* model, suggesting its potential development as a mitochondria-targeted anti-oxidant (16). 

However, the mechanism of the anti-neuroinflammatory activity of *P. australis *remains elusive. In this study, we investigated the anti-neuroinflammatory properties of *P. australis *ethanol extract (PAEE) on the regulation of pro-inflammatory mediators (nitric oxide, NO; prostaglandin E_2_, PGE_2_; inducible nitric oxide synthase, iNOS; and cyclooxygenase-2, COX-2) and pro-inflammatory cytokines (interleukin-6, IL-6 and tumor necrosis factor α, TNF-α) in LPS-stimulated BV2 microglial cells. 

## Materials and Methods

***Harvesting of P. australis and preparation of the ethanol extract***

Fresh specimens of *P. australis *were harvested from Cape Rachado located in Negeri Sembilan, West Coast of Peninsular Malaysia. Morphological identification was conducted using standard botanical approaches (26). Specimens were washed with salt water, freeze-dried (LaboGene, Brigachtal) for 48 to 72 hr at −50±2 °C, and ground into a fine powder (16). Solvent extraction was conducted using the sequential maceration method with extractants of increasing polarity. The ethanol extract (PAEE) was then concentrated *in vacuo* (LaboGene, Brigachtal), dissolved in Minimum Essential Medium Eagle (MEM) (Sigma-Aldrich, St. Louis, MO, USA, USA), and filtered-sterilized through a 0.2 µm-rated nylon membrane filter (16). 

***BV2 microglial cell culture***

The BV2 murine microglial cell line (Elabscience Biotechnology, Wuhan, Hubei, China) is an immortalized cell line that demonstrates the functional and morphological characteristics of microglia. BV2 microglial cells were maintained in Minimum Essential Medium Eagle (MEM) (Sigma-Aldrich, St. Louis, MO, USA, USA) supplemented with 10% (v/v) fetal bovine serum and 1% (v/v) penicillin-streptomycin at 37±2 °C in a 5% CO_2_-humidified incubator. BV2 cells without any treatment served as a negative control, whereas cells treated with 250 µM Nω-Nitro-L-arginine methyl ester (L-NAME) (Sigma-Aldrich, St. Louis, MO, USA, USA) served as a positive control (27).

***3-(4,5-Dimethylthiazol-2-yl)-2,5-diphenyltetrazolium bromide (MTT) cell viability assay***

The BV2 cells were plated into a 96-well plate at a density of 6.25 × 10^4^ cells per well and incubated for 24 hr at 37±2 °C in a 5% CO_2_-humidified incubator. The supernatant was discarded and replaced with fresh medium containing different concentrations of selected PAEE (0.25–8.0 mg/ml) (16) and incubated for 24 hr (designated as Experiment 2.3A) or different concentrations of LPS (0.125–8.0 µg/ml) (*Escherichia coli *O55: B5, L4524 Sigma-Aldrich, St. Louis, MO, USA) and incubated for 24 hr (designated as Experiment 2.3B).

The protective effects of PAEE against LPS-stimulated cytotoxicity in BV2 cells were studied by pre-treating the cells with a selected range of concentrations from Experiment 2.3A (0.25–2.0 mg/ml) for 2 hr followed by 1 µg/ml LPS for 24 hr (designated as Experiment 2.3C). Ten microliter of 0.5 mg/ml MTT (Merck & Co, Rahway, NJ) was added to each well and incubated for 4 hr. Supernatant was discarded, and insoluble formazan was dissolved in 100 µl dimethyl sulfoxide (DMSO). The MTT reduction was determined spectrophotometrically at 570 nm with a reference wavelength of 630 nm in a UV-Vis spectrophotometer microplate reader (Infinite 200 Pro, Männedorf, Switzerland) and expressed as a percentage of viable cells relative to the negative control.

The most optimum concentration of PAEE determined based on Experiment 2.3C (0.25–2.0 mg/ml) and 1 µg/ml LPS were selected for subsequent assays of neuroinflammation.

***Measurement of nitric oxide production***

The production of NO was measured according to the method described by Oh *et al*. (28) with minor modifications. The BV2 cells were plated into a 96-well plate at a density of 6.25 × 10^4^ cells per well and incubated for 24 hr at 37±2 °C in a 5% CO_2_-humidified incubator. The supernatant was discarded and pre-treated with fresh medium containing 0.25, 0.5, 1.0, or 2.0 mg/ml PAEE or 250 µM L-NAME for 2 hr before exposure to 1 µg/ml LPS for 24 hr. A total of 100 μl supernatant was transferred to a 96-well plate, to which was added the same volume of Griess reagent (1% [w/v] sulfanilamide and 0.1% [w/v] N-1-napthylethylenediamine dihydrochloride in 5% [v/v] phosphoric acid; Cell Signaling Technology, Danvers, MA, USA). The NO production was *determined* spectrophotometrically at *550 nm *in a UV-Vis spectrophotometer microplate reader (Infinite 200 Pro, Männedorf, Switzerland) and expressed as micromolar (µM) relative to the negative control.

***Measurement of prostaglandin E***_2_ ***production***

The BV2 microglial cells were plated into a 96-well plate at a density of 6.25 × 10^4^ cells per well and incubated for 24 hr at 37±2 °C in a 5% CO_2_-humidified incubator. The supernatant was discarded and pre-treated with fresh medium containing 0.5, 1.0, or 2.0 mg/ml PAEE for 2 hr before exposure to 1 µg/ml LPS for 24 hr. The supernatant was then subjected to PGE_2_ measurement by enzyme-linked immunosorbent assay (ELISA) according to the manufacturer’s protocol of Parameter™ Prostaglandin E_2_ Immunoassay (R&D Systems, Minneapolis, MN). The PGE_2_ production was determined spectrophotometrically at 450 nm with a reference wavelength of 570 nm in a UV-Vis spectrophotometer microplate reader (Infinite 200 Pro, Männedorf, Switzerland) and expressed as picogram per milliliter (pg/ml) relative to the negative control.

***Intracellular reactive oxygen species assay***

The intracellular ROS level was determined according to protocols described by Subermaniam *et al*. (16) with minor modifications. The BV2 microglial cells were plated into a 96-well plate at a density of 6.25 × 10^4^ cells per well and incubated for 24 hr at 37±2 °C in a 5% CO_2_-humidified incubator. The supernatant was discarded and pre-treated with fresh medium containing 0.5, 1.0, or 2.0 mg/ml PAEE for 2 hr before exposure to 1 µg/ml LPS for 24 hr. The supernatant was removed and replaced with 25 µM of 2′,7′–dichlorofluorescein diacetate (DCFH-DA) (Sigma-Aldrich, St. Louis, MO, USA), further incubated for 30 min at 37±2 °C, and washed twice with phosphate buffer saline (PBS). The fluorescence intensity was determined spectrophotometrically at an excitation wavelength of 485 nm and emission wavelength of 535 nm in a UV-Vis spectrophotometer microplate reader (Infinite 200 Pro, Männedorf, Switzerland) and expressed as a percentage relative to the negative control.

***Western blot analysis***

Western blot analysis was carried out according to the method described by Kim *et al*. (29) with minor modifications. The BV2 microglial cells were plated into a 96-well plate at a density of 6.25 × 10^4^ cells per well and incubated for 24 hr at 37±2 °C in a 5% CO_2_-humidified incubator. The supernatant was discarded and pre-treated with fresh medium containing 0.5, 1.0, or 2.0 mg/ml PAEE for 2 hr before exposure to 1 µg/ml LPS for 24 hr. The lysate was diluted in lysis buffer containing protease inhibitor cocktail (Sigma-Aldrich, St. Louis, MO, USA) and 1 mM phenylmethylsulfonyl fluoride (Calbiochem, San Diego, CA, USA). An equal amount of proteins (20 μg) was separated in 10% sodium dodecyl sulfate-polyacrylamide gel electrophoresis (SDS-PAGE) and transferred to polyvinylidene fluoride (PVDF) membranes (Amersham Biosciences, Piscataway, NJ, USA). The membranes were blocked with 5% skim milk in TBST (Tris-buffered saline in 0.1% TWEEN^®^ 20) buffer for 1 hr at room temperature and incubated with primary antibodies [rabbit (mouse-specific) monoclonal anti-iNOS antibody, Cat. No. 13120, 1:1000 dilution, or rabbit (mouse-specific) monoclonal anti-COX-2 antibody, Cat. No. 12282, 1:1000 dilution, Cell Signaling Technology, Danvers, MA, USA] in blocking buffer at 4 °C overnight in a humidity chamber.

After washing five times in TBST, the membranes were incubated with a secondary antibody (horseradish peroxidase (HRP)-conjugated) for 1 hr at room temperature. Proliferating cell nuclear antigen (PCNA; Cell Signaling Technology, Danvers, MA, USA) and β-actin (Cell Signaling Technology, Danvers, MA, USA) were employed as the loading controls. The HRP signal was detected by chemiluminescence (WesternBright™ ECL Spray, Advansta Inc, San Jose, CA, USA) and visualized using the LAS-3000 LuminoImage analyzer (Fujifilm, Tokyo, Japan). Resultant bands were quantified with Image J densitometry (National Institutes of Health and the Laboratory for Optical and Computational Instrumentation, Madison, WI, USA) and normalized to β-actin (rabbit monoclonal antibody, Cat. No. 4970, 1:1000, Cell Signaling Technology, Danvers, MA, USA).

***Measurement of pro-inflammatory cytokine secretion***

The BV2 microglial cells were plated into a 96-well plate at a density of 6.25 × 10^5^ cells per well and incubated for 24 hr at 37±2 °C in a 5% CO_2_-humidified incubator. The supernatant was discarded and pre-treated with fresh medium containing 0.5, 1.0, or 2.0 mg/ml PAEE for 2 hr before exposure to 1 µg/ml LPS for 24 hr. The supernatant was collected, and the production of TNF-α and IL-6 was measured using the Mouse TNF-α Quantikine ELISA Kit and Mouse IL-6 Quantikine ELISA Kit (R&D Systems, Minneapolis, MN, USA). The concentration of TNF-α or IL-6 was determined by measuring the absorbance at 450 nm and 570 nm as reference wavelengths in a UV-Vis spectrophotometer microplate reader and expressed as picogram per milliliter (pg/ml) relative to the negative control.

***Isolation and identification of compounds from*** ***PAEE***

**Figure 2:**
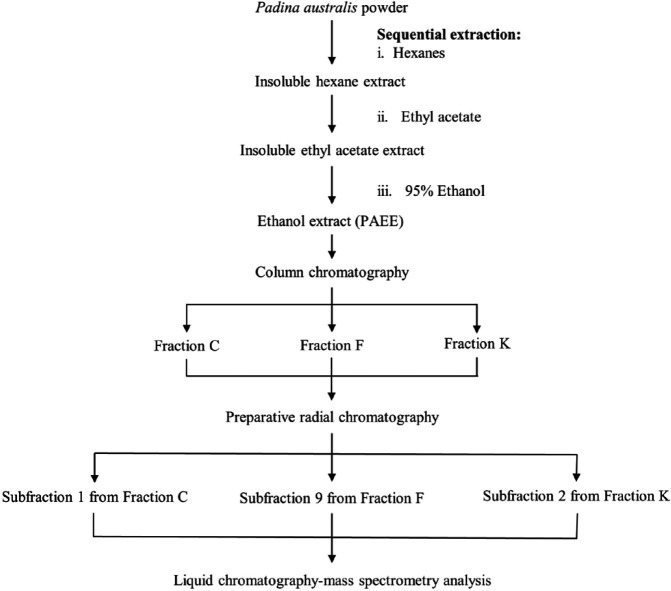
Extraction process and fractionation of *Padina australis*

PAEE was subjected to flash column chromatography (Silica gel 60, 0.04-0.06 mm; Merck, Darmstadt, Germany). The *polarity *of the eluting solvent was gradually increased starting from chloroform:hexanes (1:1) to chloroform:methanol (99:1-85:15). Eluted fractions were monitored via thin-layer chromatography (TLC) and appropriate fractions were combined into 11 main fractions (A-K). The selected combined fractions (C, F, and K) were further fractionated using preparative radial chromatography (PRC) (Silica gel 60 PF_254,_ Merck, Darmstadt, Germany) into five, nine, and seven subfractions, respectively. The solvent systems used in the PRC included chloroform:hexanes (1-2:1-3), diethyl ether:hexanes (3:1), and dichloromethane. The eluted subfractions (1^st^, 9^th^, and 2^nd^ subfractions from fractions C, F, and K, respectively) were concentrated and further analyzed by liquid chromatography-mass spectrometry (LC-MS). Figure 2 shows the workflow of the extraction process and fractionation of *P. australis*. 

The LC-MS analysis was performed on Agilent 1290 infinity liquid chromatograph (Agilent Technologies, Wilmington, DE, USA), coupled to the Agilent 6520 Accurate-Mass Q-TOF mass spectrometer with dual electrospray ionization (ESI) source. A reverse-phase high-performance liquid chromatography (HPLC) column (Agilent Eclipse XBD-C18) with a length of 150 mm and an internal diameter of 2.1 mm in a narrow-bore scale format along with a particle size of 3.5 µm was used (30). Automated mass spectrometry - mass spectrometry (aMSMS) mode was employed. Data were processed using Agilent MassHunter Qualitative Analysis software (Version B.07.00) and the Molecular Feature Extraction (MFE) small molecule algorithm. Compound identification was performed using the MassHunter METLIN Metabolite Personal Compound Database and Library (PCDL) (Agilent Technologies, Santa Clara, CA, USA).

***Statistical analysis***

Data analysis was performed using the Statistical Package for Social Sciences (SPSS, version 23.0 for Windows, Chicago, IL, USA). Data were expressed as mean ± standard deviation (SD) from three replicates. The distribution of variables (assumptions of normality) was determined using the Shapiro-Wilk test whereas Levene’s test was employed to assess the homogeneity of variance. For normally distributed data, one-way ANOVA followed by Bonferroni *post hoc* multiple comparison tests were used for equal variances assumed, whereas the Welch test followed by Games-Howell multiple comparison test were used for equal variances not assumed. For non-normally distributed data, Kruskal-Wallis one-way ANOVA (k samples) followed by pairwise multiple comparison test were used. A *P*-value of <0.05 was considered statistically significant.

## Results

***Effect of*** ***PAEE*** ***on the viability of BV2 microglial cells***

**Figure 3:**
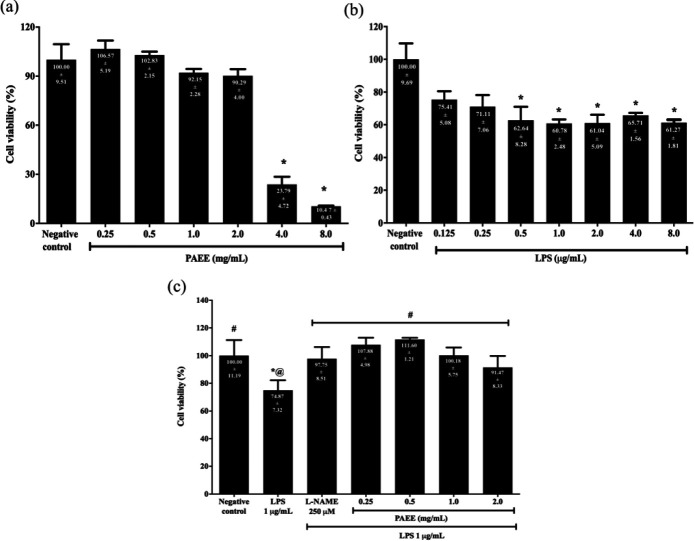
Effect of (a) PAEE and (b) LPS on the viability of BV2 microglial cells following incubation with different concentrations of PAEE and LPS for 24 hr. Asterisk (*) denotes significant difference (*P*<0.05; using Games Howell for PAEE and Kruskal-Wallis for LPS) in viability relative to the negative control. (c) Effect of PAEE on the viability of BV2 microglial cells following pretreatment with different concentrations of ethanol extract for 2 hr and exposure to 1 μg/ml of LPS for 24 hr. Asterisk (*), hash (#), and alias (@) denote a significant difference (*P*<0.05; Bonferroni) in viability relative to the negative control, LPS, and L-NAME, respectively

L-NAME: Nω-Nitro-L-argenine methyl ester; LPS: Lipopolisaccharide; PAEE: *Padina australis *ethanol extract

The effect of PAEE on the viability of BV2 microglial cells was evaluated preceding the investigation of the anti-neuroinflammatory activities of the extract to eliminate the possible cytotoxic and proliferative effects. As shown in Figure 3a, cell viability was decreased with increasing concentrations of PAEE from 1.0 to 2.0 mg/ml, but was markedly reduced at 4.0 and 8.0 mg/ml in which the viability was significantly decreased to 23.79 ± 4.72 and 10.47 ± 0.43%, respectively compared with the negative control (*P<0.05*). Considering the lower concentrations of 0.25 to 2.0 mg/ml PAEE showed no significant difference in viability compared with the negative control (*P*>0.05), the concentration range was selected for subsequent assays of viability and NO production.

***Effect of LPS on the viability of BV2 microglial cells***

The vulnerability of BV2 microglial cells to LPS-stimulated toxicity was investigated through exposure to different concentrations of LPS in the range of 0.125 to 8.0 µg/ml. Figure 3b shows that cell viability was decreased with increasing LPS concentrations. At 0.5, 1.0, 2.0, 4.0, and 8.0 µg/ml LPS, the viability was significantly decreased to 62.64 ± 8.28, 60.78 ± 2.48, 61.04 ± 5.09, 65.71 ± 1.56, and 61.27 ± 1.81%, respectively (*P<0.05*). Considering exposure to 1 µg/ml LPS caused the lowest percentage of viability at 60.78 ± 2.48, the concentration was selected for the subsequent assays of neuroinflammation.

***Effect of*** ***PAEE*** ***on the viability of BV2 microglial cells treated with LPS***

The protective effect of PAEE against 1 μg/ml LPS-stimulated cytotoxicity was investigated by pretreating BV2 microglial cells with 0.25 to 2.0 mg/ml PAEE for 2 hr followed by 1 μg/ml LPS for 24 hr. As shown in Figure 3c, 1 μg/ml LPS significantly reduced the viability to 74.87 ± 7.32% or 1.3-fold lower compared with the negative control (*P<*0.05). However, pretreatment with PAEE in the range of 0.25 to 2.0 mg/ml and L-NAME significantly increased the viability to 107.88 ± 4.98% (0.25 mg/ml), 111.60 ± 1.21% (0.5 mg/ml), 100.18 ± 5.75% (1.0 mg/ml), 91.47 ± 8.33% (2.0 mg/ml), and 97.75 ± 8.51% (L-NAME) or 1.2- to 1.5-fold higher compared with LPS (*P*<0.05). We did not observe cytotoxicity effect for any tested concentrations (0.25 to 2.0 mg/ml) (*P>*0.05). Pre-treatment with PAEE was observed to be comparable with the effect of L-NAME in increasing viability.

***Effect of*** ***PAEE*** ***on NO production in BV2 microglial cells treated with LPS***

**Figure 4:**
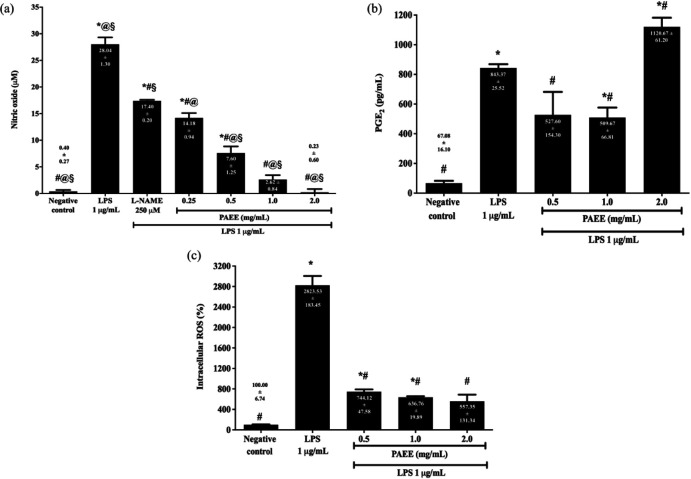
Effect of PAEE on (a) NO and (b) PGE_2_ production, and (c) intracellular ROS generation in BV2 microglial cells following pretreatment with different concentrations of PAEE for 2 hr and exposure to 1 μg/ml of LPS for 24 hr. Asterisk (*), hash (#), alias (@), and section (§) denote a significant difference [*P*<0.05; Bonferroni (NO) and Games-Howell (PGE_2_ and intracellular ROS)] in NO and PGE_2_ production, and intracellular ROS level relative to the negative control, LPS, L-NAME, and 0.25 mg/ml PAEE, respectively

L-NAME: Nω-Nitro-L-argenine methyl ester; LPS: Lipopolysaccharide; NO: Nitric oxide; PAEE: *Padina australis *ethanol extract; PGE_2_ : Prostaglandin E_2_; ROS: Reactive oxygen species

 We evaluated NO production in culture supernatants by detecting the level of nitrite using a Griess reaction.  As shown in Figure 4a, 1 µg/ml LPS significantly increased the NO production from 0.40 ± 0.27 µM to 28.04 ± 1.30 µM or 70.1-fold higher compared with the negative control (*P<*0.05). However, pretreatment with PAEE in the range of 0.25 to 2.0 mg/ml and L-NAME significantly reduced NO production to 14.18 ± 0.94 µM (0.25 mg/ml), 7.60 ± 1.25 µM (0.5 mg/ml), 2.62 ± 0.84 µM (1.0 mg/ml), 0.23 ± 0.60 µM (2.0 mg/ml), and 17.40 ± 0.20 µM (L-NAME) or 1.6- to 121.9-fold lower compared with LPS (*P*<0.05). 

Although 0.25 mg/ml PAEE suppressed NO production to 14.18 ± 0.94 µM or 2.0-fold lower compared with LPS, it exhibited a weaker inhibitory effect against NO production compared with 0.5 to 2.0 mg/ml PAEE (*P<*0.05). Thus, higher concentrations of PAEE (0.5 to 2.0 mg/ml) were selected for subsequent assays of neuroinflammation. Interestingly, the tested concentrations of PAEE exhibited 1.2- to 75.7-fold higher inhibitory activity compared with L-NAME (*P*<0.05).

***Effect of*** ***PAEE*** ***on PGE***_2_ ***production in BV2 microglial cells treated with LPS***

 COX-2 catalyzes the conversion of free arachidonic acid to prostaglandins in LPS-stimulated neuroinflammation. As shown in Figure 4b, 1 µg/ml LPS significantly increased PGE_2_ production from 67.08 ± 16.10 pg/ml to 843.37±25.52 pg/ml or 12.6-fold higher compared with the negative control (*P<*0.05). However, pretreatment with 0.5 and 1.0 mg/ml PAEE significantly suppressed the PGE_2_ production to 527.60 ± 154.30 and 509.67 ± 66.81 pg/ml or 1.6- and 1.7-fold lower compared with LPS (*P<*0.05), respectively. On the other hand, 2.0 mg/ml PAEE failed to inhibit PGE_2_ production and actually increased PGE_2_ levels to 1120.67 ± 61.20 pg/ml or 1.3-fold higher compared with LPS (*P<*0.05).

***Effect of*** ***PAEE*** ***on intracellular ROS generation in BV2 microglial cells treated with LPS***

ROS act as second messengers to amplify the inflammatory function of microglia, activate the anti-oxidant response elements, and restore redox homeostasis. As shown in Figure 4c, 1 µg/ml LPS significantly increased the intracellular ROS level from 100.00 ± 6.74% to 2823.53 ± 183.45% or 28.2-fold higher compared with the negative control (*P<*0.05). However, pretreatment with 0.5, 1.0, and 2.0 mg/ml PAEE significantly reduced ROS generation to 744.12 ± 47.58, 636.76 ± 19.89, and 557.35 ± 131.34% or 3.8- to 5.1-fold lower compared with LPS (*P<*0.05).

***Effect of*** ***PAEE*** ***on the expression of iNOS and COX-2 in BV2 microglial cells treated with LPS***

**Figure 5:**
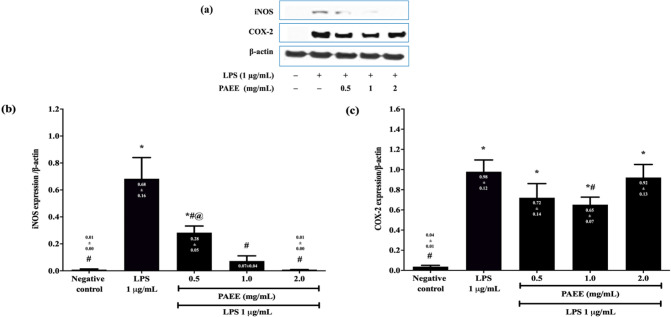
Effect of PAEE on the protein expression of iNOS and COX-2 in BV2 microglial cells following pretreatment with different concentrations of PAEE for 2 hr and exposure to 1 μg/ml of LPS for 24 hr. iNOS and COX-2 were evaluated by western blot analysis (a), the relative expression of iNOS (b), and COX-2 (c) were evaluated by densitometry with β-actin as an internal protein control. The bands corresponding to iNOS and COX-2 are noticeably more intense in LPS compared with that of negative control, whereas PAEE showed less intense bands compared with LPS. Asterisk (*) and hash (#) denote a significant difference [*P*<0.05; Games-Howell (iNOS) and Bonferroni (COX-2)] in the expression of iNOS and COX-2 relative to the negative control and LPS, respectively

COX-2: Cyclooxygenase-2; iNOS: Inducible nitric oxide synthase; LPS: Lipopolysaccharide; PAEE: *Padina australis *ethanol extract

Figure 5a shows the Western blot analysis of iNOS and COX-2 expression. Treatment with 1 µg/ml LPS significantly increased the iNOS expression from 0.01 ± 0.00 to 0.68 ± 0.16 or 68.0-fold higher compared with the negative control (*P<*0.05) (Figure 5b). However, pretreatment with 0.5, 1.0, and 2.0 mg/ml PAEE down-regulated iNOS expression in a dose-dependent manner to 0.28 ± 0.05, 0.07 ± 0.04, and 0.01 ± 0.00 or 2.4-, 9.7-, and 68.0-fold lower compared with LPS (*P<*0.05), respectively. Treatment with 1 µg/ml LPS also significantly increased COX-2 expression from 0.04 ± 0.01 to 0.98 ± 0.12 or 24.5-fold higher compared with the negative control (*P<*0.05) (Figure 5c). However, only 1.0 mg/ml PAEE significantly reduced COX-2 expression to 0.65 ± 0.07 or 1.5-fold lower compared with LPS (*P<*0.05).

***Effect of PAEE on the secretion of TNF-α and IL-6 in BV2 microglial cells treated with LPS***

**Figure 6:**
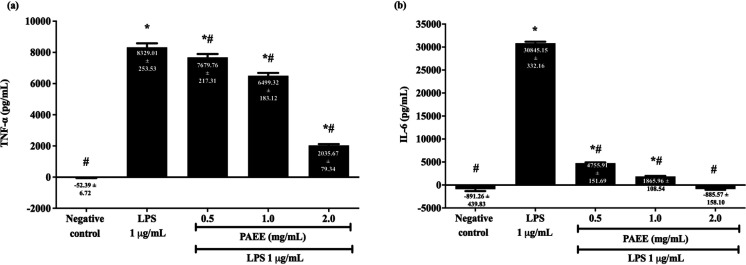
Effect of PAEE on the expressions of (a) TNF-α and (b) IL-6 in BV2 microglial cells following pretreatment with different concentrations of PAEE for 2 hr and exposure to 1 μg/ml of LPS for 24 hr. Asterisk (*) and hash (#) denote a significant difference [*P*<0.05; Bonferroni (TNF-α) and Games-Howell (IL-6)] in the TNF-α and IL-6 concentrations relative to the negative control and LPS, respectively

IL-6: Interleukin-6; LPS: Lipopolysaccharide; PAEE: *Padina australis* ethanol extract; TNF-α: Tumor necrosis factor-alpha

Activated BV2 microglial cells showed induced secretion of pro-inflammatory cytokines, including TNF-α and IL-6. As shown in Figure 6a, 1 µg/ml of LPS significantly increased the secretion of TNF-α from -52.39 ± 6.72 pg/ml to 8329.01 ± 253.53 pg/ml or 100.63% higher compared with the negative control (*P<0.0*5). However, pretreatment with 0.5, 1.0, and 2.0 mg/ml PAEE suppressed the TNF-α secretion to 7679.76 ± 217.31, 6499.32 ± 183.12, and 2035.67 ± 79.34 pg/ml, or 7.80, 21.97 and 75.56% lower compared with LPS (*P<0.0*5), respectively. As shown in Figure 6b, 1 µg/ml of LPS significantly increased the secretion of IL-6 from -891.26 ± 439.83 pg/ml to 30845.15 ± 332.16 pg/ml or 102.89% higher compared with the negative control (*P<*0.05). However, pretreatment with 0.5, 1.0, and 2.0 mg/ml PAEE markedly suppressed the IL-6 secretion to 4755.91 ± 151.69, 1865.96 ± 108.54, and -885.57 ± 158.10 pg/ml, or 84.58, 93.95 and 102.87% lower compared with LPS (*P<*0.05), respectively.

***Identification of the major compounds in PAEE fractions***

**Figure 7:**
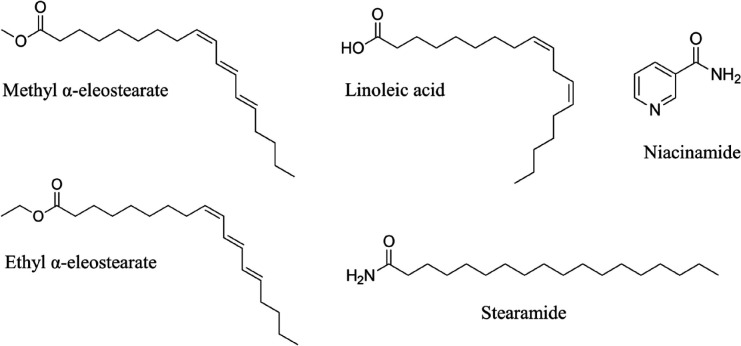
Identified compounds from *Padina australis *ethanol extract

Liquid chromatography-mass spectrometry (LC-MS/MS using aMSMS mode) analysis of subfractions C-1, F-9, and K-2 detected 93, 73, and 31 corresponding peaks in the positive ion mass spectra. A total of five major compounds were identified by searching the Metlin database using a molecular formula generator (MFG) mass score above 90% and a mass deviation [difference between observed (*m/z*) and calculated (MFG) mass] of ± 5 ppm. These compounds included two esters, and an amide (stearamide) (31) from subfraction C-1, a polyunsaturated fatty acid (PUFA) (linoleic acid) from subfraction F-9, and an amide (niacinamide, a major form of vitamin B3) (32) from subfraction K-2 (Figure 7). 

The ESIMS of subfraction C-1 shows molecular ion peaks at *m/z* 293.2471, 307.2628, and 307.2628, establishing the molecular formula of methyl α-eleostearate (C_19_H_32_O_2_), ethyl α-eleostearate (C_20_H_34_O_2_), and stearamide (C_18_H_37_NO). The ESIMS of subfraction F-9 shows molecular ion peaks at *m/z* 281.246, establishing the molecular formula of linoleic acid (C_18_H_32_O_2_). The ESIMS of subfraction K-2 shows molecular ion peaks at *m/z* 123.0531, establishing the molecular formula of niacinamide (C_6_H_6_N_2_O) (Table 1). The chromatogram and mass spectrum peak list of the five isolated compounds can be found in the supplementary file. The remaining compounds with MFG scores above the cut-off values did not match any of the known molecules in the Metlin database, which leaves a large family of minor compounds in the PAEE still to be examined.

**Table 1:** Isolated and identified compounds from *Padina australis* ethanol extract based on liquid chromatography mass spectrometry/mass spectrometry (LCMS/MS) analysis

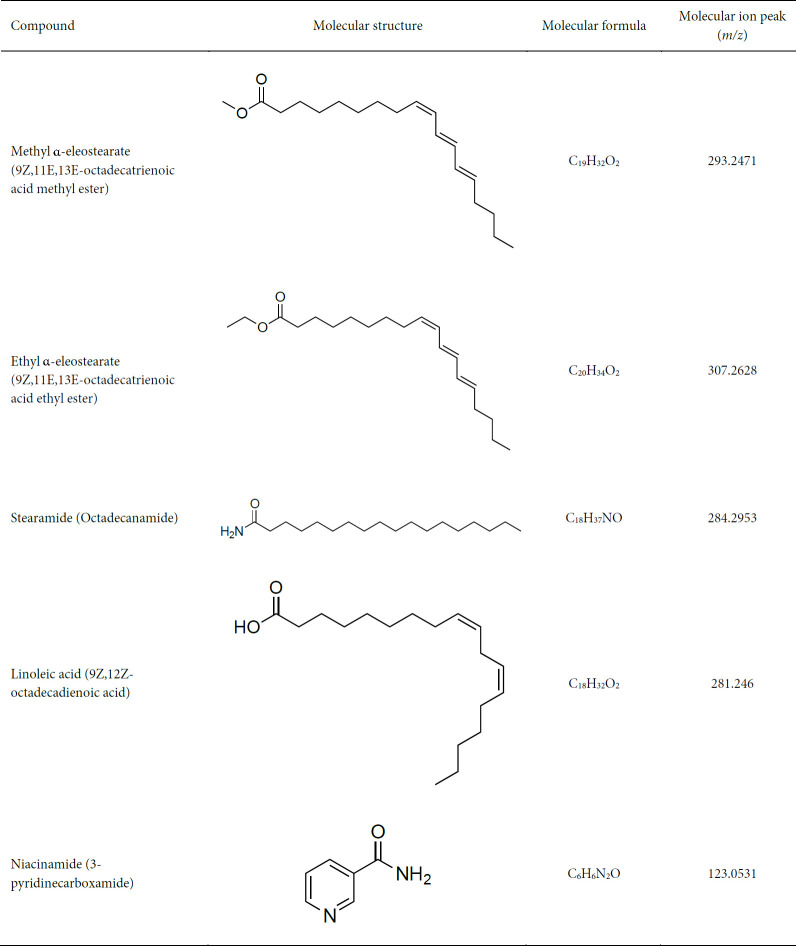

**Figure 8:**
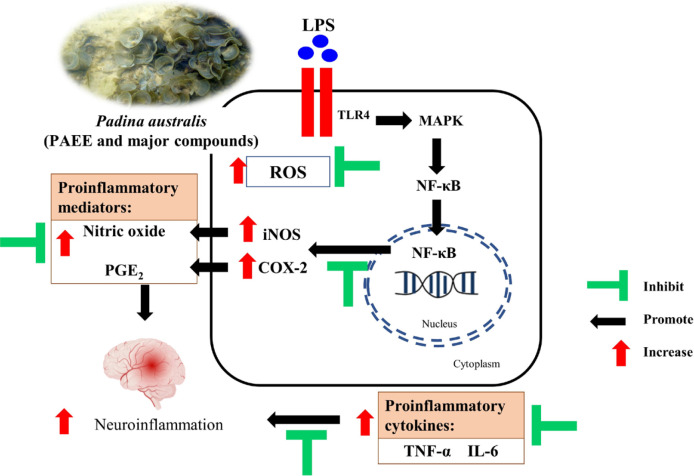
Proposed protective effects of PAEE against LPS-stimulated neuroinflammation in BV2 cells. Figure was created using BioRender (https://biorender.com/) and Microsoft PowerPoint 2017. The image of *P. australis *was captured at Cape Rachado, Port Dickson, Negeri Sembilan, Malaysia

LPS: Lipopolysaccharide; PAEE: *Padina australis* ethanol extract

## Discussion

*P. australis* has been demonstrated to attenuate oxidative damage induced by high-dose corticosterone in PC-12 cells mimicking the effects of depression due to the presence of phytochemical compounds (16), indicating a protective role of *P. australis*-based anti-oxidants in the mitochondrial defense against ROS generation. Accumulating evidence indicates that suppression of microglia-mediated neuroinflammation is a potentially effective *therapeutic target* for halting the progression of neurodegenerative diseases (6, 33, 34). 

 In this study, we extended our investigation into the anti-neuroinflammatory potential of PAEE against LPS-stimulated neuroinflammation using BV2 microglial cells as the *in vitro* model system. We are particularly interested in elucidating the mechanism underlying the anti-inflammatory potential of PAEE by studying the expression of various proteins involved in inflammatory signaling pathways.

 We observed that 1 µg/ml LPS profoundly increased the production of NO and PGE_2_ in BV2 microglial cells which is in line with previous findings (17, 28, 35-38). Conversely, pretreatment with 0.5-2.0 mg/ml PAEE significantly reduced the production of NO and PGE_2_ in BV2 microglial cells. Notably, a study by Oh *et al*. (28) showed that *Sargassum serratifolium* ethanol extract at similar concentrations to that of PAEE (0.5 to 2.0 µg/ml) also inhibited LPS-stimulated NO and PGE_2_ production in BV2 microglial cells. Moreover, we found that the PAEE treatment demonstrated a higher percentage of NO reduction compared with L-NAME, indicating PAEE is a potent inhibitor of NOS. A study by Gany *et al*. (17) observed that a low concentration of a *P. australis* dichloromethane or methanol extract (0.4 mg/ml) was able to inhibit NO production to 73.63 ± 3.81% and 75.67 ± 0.21%, respectively, which was similar to our finding that 0.5 mg/ml PAEE decreased NO production to 72.90%. Moreover, the *P. australis* methanol extract was found to inhibit PGE_2_ production by 30%, which was 7% lower than that of 0.5 mg/ml PAEE at 37%. 

 Neuroinflammation and oxidative damage are undoubtedly hallmarks of neurodegeneration characterized by the generation and accumulation of excessive free radicals, including ROS and RNS, therefore contributing to disease progression (39, 40). Importantly, neuroinflammatory response has been shown to initiate cellular events associated with redox imbalance and microglia-derived oxidant production by NADPH oxidase. An excessive generation of intracellular and extracellular ROS leads to direct cellular damage and triggers the activation of microglia and leukocytes, which in turn, causes dysregulated generation of ROS and RNS, resulting in a vicious cycle. Indeed, ROS and RNS seem to be common features linked to microglial response, which is tightly related to Parkinson’s disease (PD), Alzheimer’s disease (AD), and frontotemporal dementia (FTD) involving dysfunctional protein aggregation and protein homeostasis imbalance. Considering the complex roles of oxidative damage in neuroinflammation, the regulation of cellular ROS may represent a potential treatment to impede neurodegeneration (37).

 In this study, 1 µg/ml LPS increased intracellular ROS level, similar to the findings (36–38, 41). In contrast, pretreatment with PAEE markedly reduced ROS generation to 3.79- to 5.07-fold lower compared with LPS, indicating its protective effects against detrimental sequel of excessive accumulation of ROS by restoring redox balance via increased anti-oxidant activities (16). Overall, our *results* are very similar to those reported by Kim *et al*. (42) and others (43), who demonstrated ethanol extracts of various species of brown macroalgae including *Sargassum horneri*, *Saccharina japonica*, *Undaria pinnatifida,* *Sargassum fulvellum, Carpomitra costata* (42), and sargachromenol-enriched* Myagropsis myagroides* (43) had potent protective effects against neuroinflammation via suppressing ROS generation in BV2 microglial cells treated with LPS. 

 Consequently, NF-κB plays a key role in the regulation of microglia-mediated neuroinflammation. However, dysregulation of NF-κB has been linked to aberrant neuroinflammation through the release of pro-inflammatory mediators. Moreover, NF-κB-binding specific regions have been identified in pro-inflammatory genes such as iNOS, COX-2, TNF-α, and IL-6 (44). In particular, COX-2 triggers pro-inflammatory processes that can aggravate neuronal degeneration and functional impairments through PGE_2_ production and subsequent activation of four G-protein coupled cell surface receptors, termed EP1, EP2, EP3, and EP4 receptors. 

 In this study, 1 µg/ml LPS increased the expression of iNOS and COX-2, which is similar to findings of other studies (36–38, 43). In contrast, pretreatment with PAEE significantly suppressed protein expressions of iNOS and COX-2, resulting in concomitant reductions of NO and PGE_2_. Ethanol extracts of other species of brown macroalgae, namely *M. myagroides* (42), *S. serratifolium* (28), and *C. costata* (43) have also been demonstrated to decrease mRNA expression and protein levels of iNOS and COX-2, resulting in the inhibition of NO and PGE_2_ production in BV2 microglial cells upon exposure to LPS. 

 We also observed that 1 µg/ml LPS increased the secretion of TNF-α and IL-6, similar to the findings of other studies (17, 37, 38, 45, 46). In contrast, pretreatment with 2.0 mg/ml PAEE exhibited the most potent activity by reversing the surge in TNF-α and IL-6 by 75.56% and 102.87%, respectively, suggesting its possible role in the attenuation of elevated *level *of circulating pro-inflammatory cytokines. On the other hand, Gany *et al*. (17) demonstrated that a low dose of *P. australis* methanol extract (0.4 mg/ml) attenuated 94.45 ± 1.92% and 92.07 ± 1.99% of TNF-α and IL-6, respectively. The discrepancy in these findings may be due to genetic variations or phenolic composition of *P. australis* from different geographical locations, extraction procedure, particle size, storage conditions and time, and the presence of interfering substances in the extracts (47). Likewise, ethanol extracts of *M. myagroides* (42) and *S. serratifolium* (28) have also been observed to decrease the protein level of TNF-F-α, IL-1β, and IL-6 in BV2 microglial cells upon exposure to LPS.

 The LC-MS data revealed five compounds including esters, polyunsaturated fatty acids, and amides. Among these compounds, ethyl ɑ-eleostearate, stearamide, linoleic acid, and niacinamide have been reported to possess therapeutic properties. Ethyl ɑ-eleostearate or 9Z,11E,13E-octadecatrienoic acid ethyl ester is a conjugated PUFA derivative with anticancer properties. Yasui *et al*. (48) reported that ethyl ɑ-eleostearate can reduce viability and induce apoptosis in human colon cancer CaCo-2 cells. 

 Stearamide derived from *Channa pleurophthalma* fin waste was found to possess anti-inflammation, antipruritic, antifungal, and antimicrobial properties using the Prediction of Activity Spectra for Substances (PASS) online tool (49). Another study (50) also reported that stearamide isolated from mushrooms such as *Amanita *sp., *Cantharellus* sp., *Ganoderma lucidum,* and *Lactarius kabansus* exhibited antibacterial properties against *Salmonella typhi*. 

 On the other hand, linoleic acid is an omega-6 (⍵-6) polyunsaturated fatty acid (PUFA) that can be elongated and desaturated to form arachidonic acid, the precursor of various inflammatory cytokines (prostaglandins, leukotrienes, and endocannabinoids) (51). Collectively, ω-3 and ω-6 PUFAs, including linoleic acid, account for approximately 50% of the total *lipids* in red and brown macroalgae (52). Although preclinical evidence suggests that *excess* dietary linoleic acid increases the vulnerability to neuroinflammation (53), both ω-3 and ω-6 PUFAs have been shown to exhibit anti-inflammatory properties (54). In another study (55), pretreatment with linoleic acid for 24 hr reduced NO production and protein level of iNOS in BV2 microglial cells following exposure to LPS. Linoleic acid isolated from an ethyl acetate fraction of *Lignosus rhinocerotis* sclerotia has been found to suppress NO production and expression of iNOS and COX-2 in BV2 microglial cells (56). Moreover, researchers (57) observed that pretreatment with linoleic acid for 24 hr suppressed mRNA expression of COX-2 in BV2 microglial cells following exposure to Aβ_42_ oligomers for 1 hr but not 4 hr, indicating the short-term protective effect of linoleic acid against neuroinflammation in an AD model. 

 In addition, niacinamide has been observed to inhibit poly (ADP-ribose) polymerase 1 (PARP-1) that facilitates diverse inflammatory responses orchestrated by pro-inflammatory cytokines, chemokines, and adhesion molecules (58). The ability of niacinamide to pass rapidly and bi-directionally through the blood-brain barrier contributes to its efficacy in promoting neuroprotection (59). Furthermore, niacinamide has been found to ameliorate inflammation by reducing the mRNA expression of IL-1β and IL-6, and nuclear translocation of p-NF-κB in RAW264.7 macrophages (60). Overall, the anti-neuroinflammatory effects of *P. australis* were attributed to the presence of linoleic acid and niacinamide. 

 Taken together, our findings suggest that *P. australis* and its compounds possess protective effects against LPS-stimulated neuroinflammation in BV2 microglial cells. The proposed anti-neuroinflammatory mechanism of PAEE is presented in Figure 8, in which LPS interacts with TLR-4 leading to the activation of mitogen-activated protein kinase (MAPK) and nuclear factor-κB (NF-κB) signaling pathways. In the nucleus, activated NF-κB promotes the transcription of NF-κB-dependent genes in the regulation of expression of iNOS and COX-2, generating NO and PGE_2_, respectively. Upon exposure to 1 μg/ml LPS, the anti-oxidant substances (16) and major compound of PAEE attenuated excessive generation of intracellular ROS, pro-inflammatory mediators (NO, PGE_2_, iNOS, and COX-2), and pro-inflammatory cytokines (TNF-α and IL-6). Taken together, our findings suggest that *P. australis* and its potential compounds possessed protective effects against LPS-stimulated neuroinflammation in BV2 microglial cells.

## Conclusion

Our current study demonstrated PAEE has protective effects against LPS-stimulated neuroinflammation in BV2 microglial cells by suppressing the excessive generation of intracellular ROS, and pro-inflammatory mediators and cytokines. These effects can be attributed to major compounds such as linoleic acid and niacinamide. The anti-neuroinflammatory and anti-oxidant properties of *P. australis* may lead to the development of novel therapeutic strategies for neurodegenerative diseases. 

## Authors’ Contributions

KS and KHW conceived the study; KS and SYL prepared the original draft; YYY, SHL, WSY, LWL, and KHW reviewed and edited the manuscript; KS, SYL, and LWL helped with visualization and illustration; YYY, SHL, and KHW supervised the study. All authors have agreed to the contents and approved the final version for publication.

## Conflicts of Interest

None.
